# Does regular strength training cause urinary incontinence in overweight inactive women? A randomized controlled trial

**DOI:** 10.1007/s00192-021-04739-5

**Published:** 2021-03-15

**Authors:** Kari Bø, Lene Anette H. Haakstad, Gøran Paulsen, Anne Mette Rustaden

**Affiliations:** 1 Department of Sports Medicine, Norwegian School of Sport Sciences, PB 4014, Ullevål stadion, 0806 Oslo, Norway; 2 Department of Physical Performance, Norwegian School of Sport Sciences, Oslo, Norway; 3 Inland Norway University of Applied Science, Hamar, Norway

**Keywords:** Pelvic floor, Resistance training, Strength training, Urinary incontinence, Women’s health

## Abstract

**Introduction and hypothesis:**

Urinary incontinence (UI) is common in women who exercise. We aimed to investigate new onset UI in formerly inactive, overweight or obese women (BMI > 25) participating in three different strength training modalities compared with a non-exercising control group.

**Methods:**

This was a secondary analysis of an assessor blinded randomized controlled trial investigating the effect of 12 weeks of three strength training concepts for women on muscle strength and body composition. None of the programs included pelvic floor muscle training. International Consensus on Incontinence Questionnaire Urinary Incontinence Short Form (ICIQ-UI-SF) was used to investigate primary outcome; new onset UI, and secondary outcome; ICIQ-UI-SF sum score. Suissa and Shuster’s exact unconditional test was used to analyze difference in new onset UI. Difference in ICIQ-UI-SF sum score is presented as mean with 95% CI.

**Results:**

At baseline 40 out of 128 (31.2%) participants reported UI. Three out of 27, 2 out of 17, 2 out of 23, and 0 out of 21 women in the three training and control groups respectively had new onset UI. There were no statistically significant differences in new onset UI across the groups or when collapsing new onset UI in the intervention groups compared with the controls (7 out of 67 vs 0 out of 21), *p* = 0.124. After the intervention the control group reported worse ICIQ-UI-SF sum score than any of the training groups; mean difference − 6.6 (95% CI: −11.9, −1.27), *p* = 0.012, but there was no difference in change from baseline to 12 weeks between the groups *p* = 0.145).

**Conclusions:**

There was no statistically significant change in UI after strength training.

## Introduction

Physical activity is an important and modifiable health factor for all age groups [1, 2]. Today, regular strength training is recommended to maintain and improve function and activity of daily living and to reduce and stabilize body weight [3]. Recommendations for strength training for the general population involve performance of three sets of 8–12 close to maximum contractions of the major muscle groups 2–3 times per week [3]. The same amount of whole-body strength training is recommended for overweight and obese persons for weight reduction [4]. However, strenuous work and exercise such as weightlifting increases the intra-abdominal pressure [5] and has been listed as a possible risk factor for development of pelvic floor dysfunction (PFD) such as urinary incontinence (UI), anal incontinence, and pelvic organ prolapse (POP) in women [6–8].

Established risk factors for UI are pregnancy, age, obesity, parity, and mode of delivery, with vaginal birth being the most significant [6]. However, the very definition of SUI highlights that the condition occurs during exercise and strenuous activities [9]. Exercising women, and young, nulliparous female elite athletes also report a high prevalence of UI, especially in sports involving high-impact activities such as running and jumping [8, 10, 11]. Several authors have reported that women with SUI change their movement and exercise pattern and often drop out of regular exercise [12, 13]. To date, there is scant knowledge on female athletes participating in strength training [14], and there is little knowledge on women participating in strength training for fitness and health benefits [8]. According to the two opposite hypotheses on exercise and the pelvic floor [8], general strength training may strengthen the PFM via a co-contraction of the PFM and thereby potentially improve or prevent UI, or weaken the PFM, causing UI owing to repeated increases in intra-abdominal pressure and inadequate counteraction to keep the urethra in a positive pressure zone.

Most studies on UI in physically active women are cross sectional, and Bø and Nygaard [8] emphasized the need for prospective studies and preferably randomized controlled trials (RCTs) to investigate the effect of regular exercise on PFDs such as UI. Given the benefits of regular physical activity and specific recommendations for strength training, the effect of strength training on UI in former inactive overweight and obese women who start exercising is of special interest. The aim of the present RCT was to investigate whether participation in 12 weeks of strength training in formerly inactive, overweight or obese women causes new onset of UI. A further aim was to investigate differences in changes in the International Consensus on Incontinence Questionnaire Urinary Incontinence Short Form (ICIQ-UI-SF) sum score between the groups.

## Materials and methods

### Design

This is a convenience secondary analysis of a four-arm, parallel group, RCT. The main study was aimed at comparing the effect of three different strength training concepts with a nontreated control group on muscle strength (one repetition maximum of squat and bench press) and body composition (BMI and Inbody720) [15]. The randomization was concealed, and the assessor was blinded to group allocation. Further details of the randomization procedure can be obtained from the main study [15]. The present study evaluated the effect of these three strength training concepts compared with the control group on new onset UI.

### Ethical approval

The main study was approved by the Regional Committee for Medical Research Ethics, Oslo, Norway (REK 2012/783) and all participants signed a written consent statement before entering the study. The procedures followed the World Medical Association Declaration of Helsinki, and the RCT is listed at ClinicalTrials.gov (NCT01993953). Additional ethical approval was given for the present secondary analysis of the study (REK sør-øst D 118368). Methods, definitions, and units conform to the standards jointly recommended by the International Urogynecological Association and the International Continence Society, except where specifically noted [9].

### Participants

**Fig. 1:**
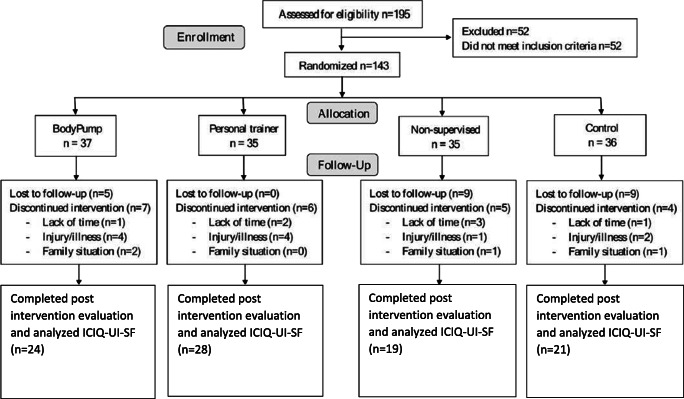
Flow chart of participants throughout the study period

One-hundred and forty-three inactive, overweight (BMI ≥ 25) or obese (BMI ≥ 30) women were randomized to 12 weeks of either BodyPump (*n* = 37), traditional heavy load strength training with a personal trainer (*n* = 35), nonsupervised traditional heavy load strength training (*n* = 35), or a non-exercising control group (*n* = 36). Inactive was defined as not meeting physical activity guidelines; performing regular structured exercise at least twice a week during the last 6 months [15]. Inclusion criteria were BMI ≥ 25, age 18–65 years old, and being inactive. Exclusion criteria were not planning absence from training during the intervention period, pregnancy or planned pregnancy, diseases/injuries contraindicated for one repetition maximal (RM) testing and heavy load resistance training including sciatica, low back pain, osteoarthritis, osteoporosis, secondary hypertension, history of coronary heart disease, stroke, arrhythmia, type I diabetes, neurological disease, obesity surgery, and psychiatric diseases [15]. Flow chart of the participants throughout the study period is shown in Fig.1. Sample size calculation was based on the primary outcome of the RCT only. With an expected difference in maximal muscle strength (squats and bench press) of 11% (effect size 0.7) between the intervention and inactive groups and a standard deviation of 15, alpha of 5%, power of 80%, and a calculated dropout of 10–20%, a minimum of 35 participants were included in each group [15].

### Intervention/exposure

All exercise groups were scheduled for three sessions of 1 h per week of strength training. Women randomized to BodyPump exercised in a group setting with an instructor, following a BodyPump program pre-choreographed by LesMills International. Each participant used a weight bar (1.25 kg), plates (1, 2.5, or 5 kg), and a step. All BodyPump sessions lasted 55 min and included between 800 and 1,000 repetitions (50–100 in each muscle group). All sessions included nine music tracks (4–6 min), each involving strength exercises for specific body parts. The following exercises were included during each session: squat, bench press, rowing, stiff-legged dead lift, clean and power press, French press, triceps press, pullover and overhead triceps press, biceps curl, lunges, squat jump, push-up, lateral raise, shoulder press, sit-ups, sit-ups to the side, and side plank [15].

The two other strength training groups followed a nonlinear periodization program, based on the same basic exercises as described for the BodyPump group. Repetitions varied between 3 and 15, and series between 2 and 4. One of the groups exercised with a personal trainer at all sessions, whereas the other group exercised nonsupervised, except for the first exercise session and a follow-up session after 6 weeks (nonsupervised group). During these two sessions the instructor guided the participants in the correct technique and advised on proper weight increase. The weights were self-selected in all exercise groups, but the participants were encouraged to perform RM. None of the exercise programs included pelvic floor muscle training (PFMT) and none of the women received any treatment for UI.

Before and after the intervention, the women answered a questionnaire on background variables, including educational level, physical activity, nutrition, smoking habits, perceived health, quality of life (QoL), motivation for exercise, musculoskeletal pain, and UI [15]. Adherence to the three strength training concepts was registered in a training diary and reported as number of sessions/percentages of 36 possible sessions.

### Outcome

The International Consensus on Incontinence Questionnaire Urinary Incontinence Short Form (ICIQ-UI-SF) [16] was included in the electronic questionnaire and provided data on frequency of UI, amount, and bother of UI at baseline and following 12 weeks of strength training. The questionnaire contains four questions where the responses to the first three questions are summarized into the ICIQ sum score. Women were categorized as continent if they answered “never” to the question: “How often do you leak urine?” (response categories: never, about once a week or less often, two or three times a week, about once a day, several times a day, all the time). The ICIQ-UI-SF categorizes amount of leakage as: none, a small amount, a moderate amount, a large amount. How much UI affects everyday life is registered on a scale from 0 to 10. Total ICIQ score is the sum of how often the women report leakage, amount of leakage, and how much the condition interferes with everyday life (maximum score: 21) [16].

The fourth question diagnoses the different subtypes of UI. Women are classified with SUI if they answer: “leaks when you cough or sneeze” and/or “leaks when you are physically active/exercising” to the question “When does urine leak occur?” Urge urinary incontinence is diagnosed if the woman responds that she leaks before she can reach the toilet and mixed urinary incontinence a combination of the above-mentioned three categories. Reliability of the ICIQ-UI-SF has been found to be good, with “moderate” to “very good” stability in test–retest analysis, and a Cronbach’s alpha of 0.95 [16]. ICIQ-UI-SF can discriminate among different groups of individuals, indicating good construct validity. Convergent validity has also been found to be acceptable, with most items demonstrating “moderate” to “strong” agreement with other questionnaires [16].

Primary outcome was new onset UI. Secondary outcome was change in ICIQ sum score between groups.

### Statistical analyses

Background variables are reported as numbers with percentages or means with standard deviations (SD). Difference in background variables are analyzed using Chi-squared test or analysis of variance (ANOVA). Prevalence is reported as numbers and percentages of each intervention group and comparisons between groups are analyzed using Chi-squared or Fisher’s exact test. Difference in adherence was assessed using ANOVA and group comparison of adherence using Tukey’s post-hoc test. Suissa and Shuster’s exact unconditional test was used to analyze difference in new onset UI between each of the strength training groups compared with the control group and when collapsing new onset UI in all three intervention groups compared with the control group [17]. Difference in total ICIQ-UI score is presented as mean with 95% confidence intervals (CIs). *p* value was set to <0.05.

## Results

Background variables of the four groups are presented in Table 1. Mean age of participants collapsed was 40 years (SD 11.0) and mean BMI was 31.0 kg/m^2^ (SD 5.0). There were no statistically significant differences between the four groups in age, BMI, number of women with parity, smokers, or educational level at baseline.

**Table 1:** Background characteristics of the participants

	BodyPump *N* = 35	Personal trainer *N* = 32	Nonsupervised *N* = 30	Control *N* = 31	*p* value
Age (years)	39 (±11)	39 (±10)	41 (±10)	41 (±11)	0.699
BMI (kg/m^2^)	30 (±5)	32 (±6)	32 (±5)	32 (±5)	0.715
Parous					
Yes	21 (60)	15 (47)	18 (60)	20 (65)	0.519
No	14 (40)	17 (53)	12 (40)	11 (35)	
Smoking					
Daily	9 (27)	2 (6)	4 (14)	4 (14)	0.212
Sometimes	0 (0)	1 (3)	1 (3)	1 (3)	
Marital status					
Married/cohabitant	20 (57)	16 (50)	17 (57)	19 (62)	0.812
Educational level					
Elementary school	1 (3)	0 (0)	1 (3)	1 (3)	0.560
High school	8 (23)	8 (25)	7 (23)	8 (26)	
University ≤4 years	15 (43)	9 (28)	15 (50)	14 (45)	
University ≥4 years	11 (31)	14 (44)	7 (23)	6 (19)	
Other education	0 (0)	1 (3)	0 (0)	2 (7)	

*N* = 128. Means with standard deviation (SD) and frequency with percentages (%)

Prevalence of UI at baseline for the total sample was 31.2% (40 out of 128 women). Number and percentage with UI in each group was 22.8% (8 out of 35), 46.9% (15 out of 32), 23.3% (7 out of 30), and 32.2% (10 out of 31), in BodyPump, personal trainer, nonsupervised, and control groups respectively. There was no statistically significant difference in prevalence between groups at baseline (*p* = 0.129).

Loss to follow-up was 11 (31.4%), 5 (15.6%), 11 (36.7%), and 10 (32.2%) in the BodyPump, personal trainer, nonsupervised, and control groups respectively (*p* < 0.001). Adherence to the different exercise programs was 54% (SD 20) in the BodyPump group, 83% (SD 15) in the personal trainer group, and 69% (SD 20) in the nonsupervised group (p < 0.001). As shown in Table 2, the BodyPump group had the lowest and the personal trainer the highest adherence.

**Table 2:** Comparison of adherence in number and percentage of sessions of a maximum of 36 sessions between personal trainer (PT), BodyPump (BP), and nonsupervised (NS) groups

Variable	Group comparison	Mean difference	*p* value*
Number of sessions	PT vs BP	11.0 ± 2.1	<0.001
	PT vs NS	5.2 ± 2.0	0.031
	NS vs BP	5.7 ± 2.2	0.034
Percentage adherence	PT vs BP	28.5 ± 5.4	<0.001
	PT vs NS	13.8 ± 5.3	0.031
	NS vs BP	14.7 ± 5.9	0.041

*Tukey’s post-hoc test

Of the 143 enrolled participants, 128 (89.5%) responded to the ICIQ-UI-SF at both baseline and at 12 weeks. Table 3 shows the prevalence of UI, reported amount of leakage, how UI affected activities of daily living, and total ICIQ-UI-SF score at baseline and after the 12-week intervention period in women completing the program. There were no statistically significant differences between groups in any items at baseline.

**Table 3:** Prevalence, amount of urinary incontinence, and how much the condition affects the daily life of completers (responding to the ICIQ-UI-SF questionnaire before and after the intervention) in the BodyPump (BP) group, personal trainer group (PT), nonsupervised group (NS), and control group ©)

Variable	BP		PT		NS		C		p value	
	Baseline	12 weeks	Baseline	12 weeks	Baseline	12 weeks	Baseline	12 weeks	Baseline	12 weeks
Prevalence: how often do you leak urine? n (%)	*N* = 23	*N* = 24	*N* = 28	*N* = 28	*N* = 19	*N* = 19	*N* = 20	*N* = 21	0.064	0.393
Never	19 (83)	19 (83)	16 (57)	17 (61)	16 (84)	14 (74)	14 (70)	13 (62)		
About once a week or less	4 (17)	4 (17)	8 (28)	7 (25)	2 (11)	5 (26)	2 (10)	3 (14)		
2–3 times a week	0 (0)	0 (0)	3 (11)	3 (11)	1 (5)	0 (0)	0 (0)	3 (14)		
About once a day	0 (0)	0 (0)	0 (0)	1 (4)	0 (0)	0 (0)	2 (10)	1 (5)		
Several times a day	0 (0)	0 (0)	1 (4)	0 (0)	0 (0)	0 (0)	2 (10)	1 (5)		
All the time	0 (0)	0 (0)	0 (0)	0 (0)	0 (0)	0 (0)	0 (0)	0 (0)		
Amount: how much urine do you leak? n (%)	*N* = 23	*N* = 22	*N* = 28	*N* = 27	*N* = 19	*N* = 19	*N* = 20	*N* = 19	0.174	0.263
None	19 (83)	18 (82)	17 (61)	16 (59)	16 (84)	15 (79)	13 (65)	10 (56)		
A small amount	4 (17)	4 (18)	11 (39)	11 (41)	3 (16)	4 (21)	7 (35)	7 (39)		
A moderate amount	0 (0)	0 (0)	0 (0)	0 (0)	0 (0)	0 (0)	0 (0)	1 (6)		
A large amount	0 (0)	0 (0)	0 (0)	0 (0)	0 (0)	0 (0)	0 (0)	0 (0)		
Affect: how much does your UI affect your life? n ± SD	*N* = 5	*N* = 5	*N* = 12	*N* = 12	*N* = 3	*N* = 3	*N* = 6	*N* = 6	0.556	0.111
(0–10)	2.2 (±1.6)	1.2 (±1.7)	2.1 (±2.3)	2.3 (±1.7)	1.3 (±0.6)	2.0 (±1.0)	3.5 (±3.3)	4.3 (±3.1)		
When do you leak urine? n (%)	*N* = 23	*N* = 22	*N* = 28	*N* = 26	*N* = 19	*N* = 19	*N* = 20	*N* = 20	0.849	0.601
Never	17 (74)	18 (82)	16 (57)	15 (57)	14 (74)	15 (79)	13 (65)	12 (60)		
Before you can get to the toilet	1 (4)	0 (0)	2 (7)	1 (4)	0 (0)	1 (5)	2 (10)	2 (10)		
When you cough or sneeze	4 (18)	4 (18)	8 (28)	8 (31)	5 (26)	3 (16)	5 (25)	6 (30)		
When you are asleep	0 (0)	0 (0)	0 (0)	0 (31)	0 (0)	0 (0)	0 (0)	0 (0)		
When you are physically active/exercising	1 (4)	0 (0)	1 (4)	1 (4)	0 (0)	0 (0)	0 (0)	0 (0)		
Have finished urinating and are dressed	0 (0)	0 (0)	0 (0)	0 (0)	0 (0)	0 (0)	0 (0)	0 (0)		
Leaks for no obvious reason	0 (0)	0 (0)	0 (0)	1 (4)	0 (0)	0 (0)	0 (0)	0 (0)		
Leaks all the time	0 (0)	0 (0)	1 (4)	0 (0)	0 (0)	0 (0)	0 (0)	0 (0)		
ICIQ score (0–21) ^a^	5.2 (±1.6, 3.2–7.2)	2.4 (±3.4, −1.8–6.6)	5.4 (±2.6, 3.7–7.1)	5.2 (±2.8, 3.5–7.0)	4.7 (±1.1, 1.8–7.5)	5.0 (±1.0, 2.5–7.5)	8.2 (±4.5, 3.4–13.0)	9.0 (±1.7, 4.6–13.4)	0.232	0.019

^a^Presented for those reporting UI

### Primary outcome

Table 3 shows no statistically significant difference between groups in the number of women with UI, amount of leakage, how much UI affected quality of life, or when urine leaked 12 weeks after the intervention. In 1 woman in the personal trainer group and 1 in the control group frequency of leakage increased from “about once a week or less often” to “two or three times per week” on the ICIQ-UI-SF.

Three out of 27, 2 out of 17, 2 out of 23, and 0 out of 21 women in the BodyPump, personal trainer, nonsupervised training, and control groups respectively had new onset UI during the 12-week training period. There were no statistically significant differences in new onset UI between each of the training groups and the control group (BodyPump vs control *p* = 0.15, personal trainer vs control *p* = 0.14, and nonsupervised vs control *p* = 0.22) or when collapsing new onset UI in the three intervention groups compared with the control group (7 out of 67 vs 0 out of 21, *p* = 0.124).

### Secondary outcome

The ICIQ-UI-SF is presented for those reporting UI. At 12 weeks, there was a statistically significant difference between groups in ICIQ-UI-SF sum score (Table 3). The score was highest in the control group, reaching statistical significance in multiple comparison testing between the control group vs BodyPump only; mean difference − 6.6 (95% CI: −11.9, −1.27), *p* = 0.012. The difference in change in ICIQ-UI-SF total score from baseline to 12 weeks across the four groups was not statistically significant (*p* = 0.145).

## Discussion

This secondary analysis of an assessor-blinded RCT evaluated the effect of performance of regular exercise training, in this case strength training, on UI in a formerly inactive female population. There was no statistically significant difference in prevalence of UI at baseline or new onset of UI after 3 months of exercise training between women participating in three different modalities of popular strength training concepts and the control group. The control group reported statistically significantly worse ICIQ-UI-SF sum score after 12 weeks than the BodyPump group, but there was no difference in change of sum score from baseline to 12 weeks between groups.

The prevalence of UI in the study population was about 31%. This is within the expected range of prevalence in this age group [6]. Overweight and obesity are risk factors for increased intra-abdominal pressure and UI [6, 18]. However, the prevalence of UI in the present study was lower than what has been found in other studies of overweight and obese women [6, 18]. A few research groups have investigated the prevalence of UI in women attending gyms and fitness centers and it varies. Fozzatti et al. [19] found that 24.6% of nulliparous women attending gyms vs 14.3% of controls reported UI, and McKenzie et al. [20] reported that 49.3% of women attending gyms and exercise classes had UI. Haakstad et al. [21] found a prevalence of UI of 16.8% of women, mean age 34.3 years (SD 10.0), who were commencing fitness club training in Oslo, Norway. Interestingly, in a study of group fitness instructors, 26% reported experiencing UI, and the prevalence was the same in instructors teaching Pilates and yoga classes [22].

In contradiction to the above-mentioned studies, large epidemiological studies have found that women who do low impact activities, mainly walking, are at a lower risk of UI [6, 8]. However, these studies are cross sectional and hampered by selection bias, as we know that women with UI withdraw from regular training [12, 13]. Hence, it is impossible to know whether exercise makes them dry or if they can exercise because they are dry. Another possible explanation of a positive association between participation in regular physical activity and continence may be that obesity is associated with UI, and women who exercise are less prone to being overweight or obese [6]. The present RCT targeted overweight and obese formerly inactive women, a group that is encouraged to do strength training to lose weight [4], and the primary aim of the study was to investigate whether three forms of strength training could increase strength and improve body composition. None of the strength training programs showed any effect on body composition [15]; hence, this was not controlled for in the present study.

Our literature search revealed no other RCTs investigating whether general exercise can lead to or worsen UI, and only two prospective studies on exercise and onset of UI were found. Larsen and Yavorek [23] investigated whether participation in a 6-week US military summer camp had an effect of UI and POP. A subgroup of 37 female paratroopers were significantly more likely to have stage II prolapse (RR = 2.72) and worsening of their pelvic support regardless of initial prolapse stage (RR = 1.57), but there was no change in UI. Another cohort study followed 125 female beginner recreational exercisers in a fitness club setting and found no change in prevalence in UI after 12 months [21]. The study populations and exercise programs of these former studies are not directly comparable with our study, but our results, including a group of inactive and overweight/obese women, confer with their results, showing no increase in UI after an exercise period. However, our results must be interpreted with caution owing to a high loss to follow-up. Unfortunately, we have no data on whether drop-out was due to UI. Several studies have found that women with UI drop out of exercise training [12, 13, 24]. However, the drop-out among the exercisers in the present study was like the drop-out from the control group with no exercise. A high drop-out is unfortunate in any RCT, but it is common in formerly inactive groups who commence an exercise program. A loss to follow-up of 27% was found in a recently published study of women commencing fitness club training in the same geographical area [21]. In the present study, a significantly lower drop-out and higher adherence was found in the personal trainer group. This group also had the heaviest workload [15]. Nevertheless, there were no differences in the prevalence of new onset UI between the personal trainer group and the other groups. There was a statistically significant difference in ICIQ total score between groups after the intervention, with the control group having a higher score than the BodyPump group, but this difference disappeared when comparing change in the score between groups. Hence, this supports the fact that there was no negative effect of strength training on UI in the present study. UI does not seem to have massively worsened in this population of overweight and obese inactive women and is therefore unlikely to have caused the drop-out and low adherence.

New onset of UI was low, and nonsignificant results may be due to type II error. The very definition of SUI implies that the condition occurs during physical exertion and increases under intra-abdominal pressure [9]. Being inactive, e.g., staying in a sitting position or moving slowly with no increase in intra-abdominal pressure or ground reaction forces, does not provoke SUI [25]. Hence, physical activity may only unmask an underlying condition and not cause it [8]. On the other hand, strenuous activity may open the levator hiatus and cause vaginal descent [8, 26]. Ree et al. [27] found that 90 min of strenuous strength and endurance training caused reduced maximum voluntary contraction of the PFM in young nulliparous women with SUI. Middlekauff et al. [26] found reduced resting pressure after 25 min of both cross-fit and walking activities. The relationship between these mechanical factors and UI during physical activity needs further investigation.

It is an important point that none of the strength training programs in the present study included PFMT and the women did not receive PFMT or other treatments for UI during the intervention period. We have no data on former treatment for UI. As PFMT has level 1 evidence and recommendation A to be effective in treatment for UI and POP [28] PFMT would have diluted a possible effect of general strength training on UI. As many women attend dance and aerobic classes at fitness clubs, this can be considered an important arena for prevention and treatment of UI if the instruction of PFMT follows general strength training principles and protocols that have been shown to be effective [3, 28]. However, Neels et al. [29] reported that only 7.1% of pregnant women had received information about the PFM before pregnancy, e.g., during yoga or Pilates classes, back school, or sporting activities. This confers with a recent study among new members from 25 fitness clubs in Oslo, Norway, in which less than 8% had received any information about PFMT by the fitness club staff [21]. If the instructors learn how to teach PFMT correctly, general exercise classes have the potential to become an important arena for PFMT. However, the effect of PFMT within a fitness class setting needs to be investigated in future large-scale RCTs.

Strengths of the present study were the RCT design, including a control group with no exercise, comparison of three commonly advocated strength training modalities for women including no PFMT, use of a reliable and valid instrument to assess UI, a blinded investigator for UI and group allocation, and close follow-up by trained instructors in the intervention groups. Limitations of the study were high drop-out and low adherence, leading to a small sample size in each of the comparison groups. A trend toward higher new onset UI in exercising women did not reach statistical significance, which may be due to small sample size. However, the trend was not supported when comparing ICIQ-UI-SF sum score changes between exercising groups and the control group during the 12-week intervention period where the control group scored worse than one of the intervention groups after the interventions. The RCT included formerly inactive and overweight or obese women and the results can only be generalized to this group, and not to female elite athletes who perform more strenuous and higher load exercise training. Regular physical activity is an important health factor, and all women, including those overweight and obese, should be encouraged to participate in fitness classes. The results of the present study indicate that general strength training does not cause UI. Owing to the small sample size our study can be considered a pilot study and serve as a basis for future sample size calculations. Further large-scale RCTs including women of different fitness levels and evaluation of start and performance of more strenuous activities, e.g., marathon running, triathlon, and cross-fit are warranted.
